# Supplementary material for: Losses resulting from deliberate exploration trigger beta oscillations in frontal cortex
Source: Front Neurosci. 2023 May 11;17:1152926. doi: 10.3389/fnins.2023.1152926 (PMC10211346; doi:10.3389/fnins.2023.1152926)
Supplement: Supplementary file 1 [file Data_Sheet_1.PDF]

Supplementary Material

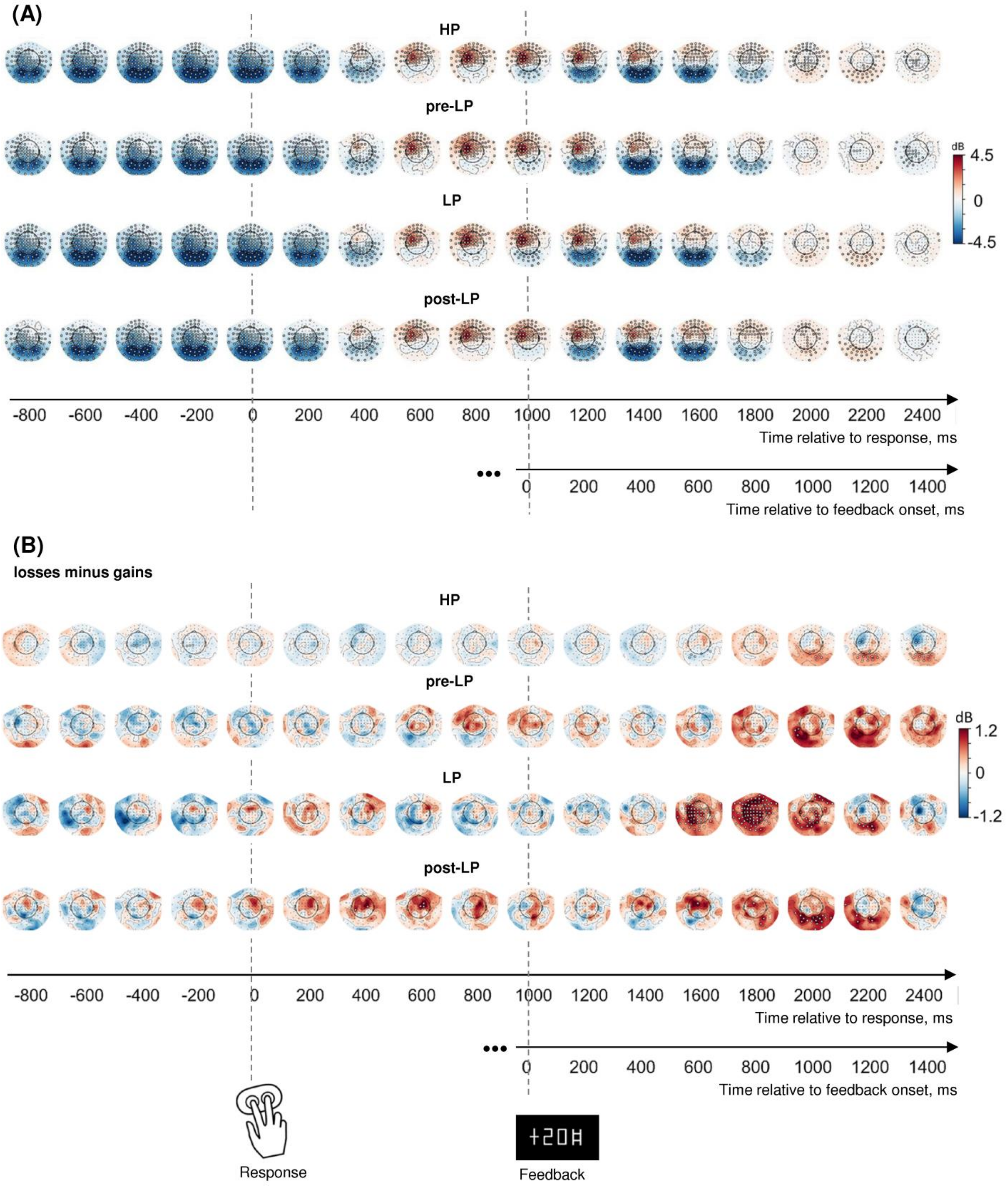

Supplementary Figure S1. Sequences of topographic maps of the power of β-band oscillations in response-locked data; significant sensors are indicated by open circles. Each map represents β power averaged over a 200-ms time window (-100 – 100 ms relative to the timestamps indicated).

(A) Each choice type minus baseline (t-statistics, uncorrected);

(B) 'Losses' minus 'gains', for each choice type (LMEM statistics, FDR-corrected for 17 time windows x 102 sensors).

Decision-related β power (-900 – -300 ms before a response onset)

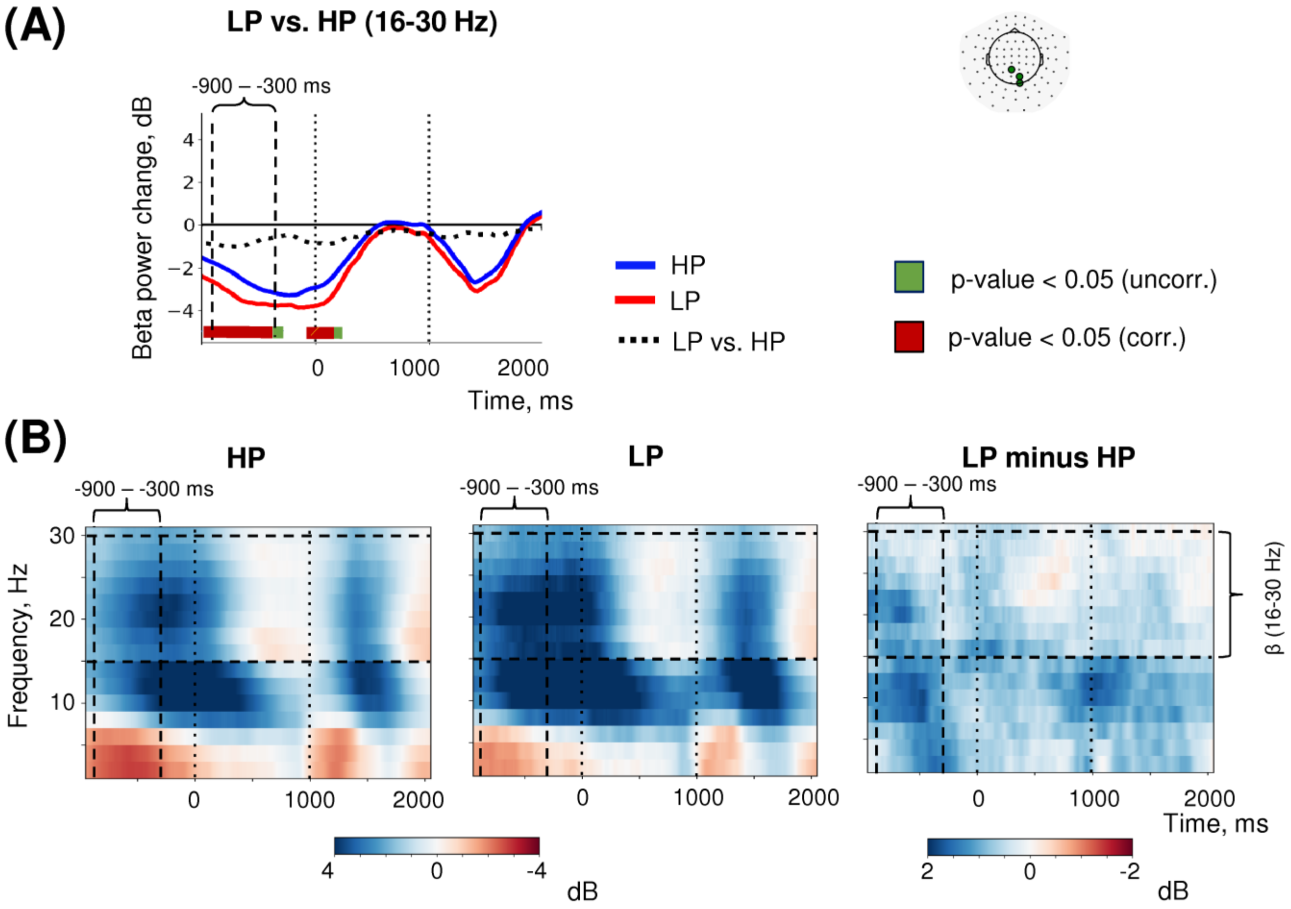

Supplementary Figure S2. Additional time-frequency analysis for the decision-related effect.

(A) Timecourses of β-band power averaged over three most significant sensors (shown in the inset at the top). Each graph represents two timecourses and the difference between them. Lines at the bottom of each graph indicate significance of the difference (t-test, $p < 0.05$; green: uncorrected; brown: FDR-corrected over time points).

(B) Event-related spectral perturbations of MEG power averaged within the same three sensors obtained in the main LMM analysis.

'0' on the timeline corresponds to response, and '1000' – to feedback onset.

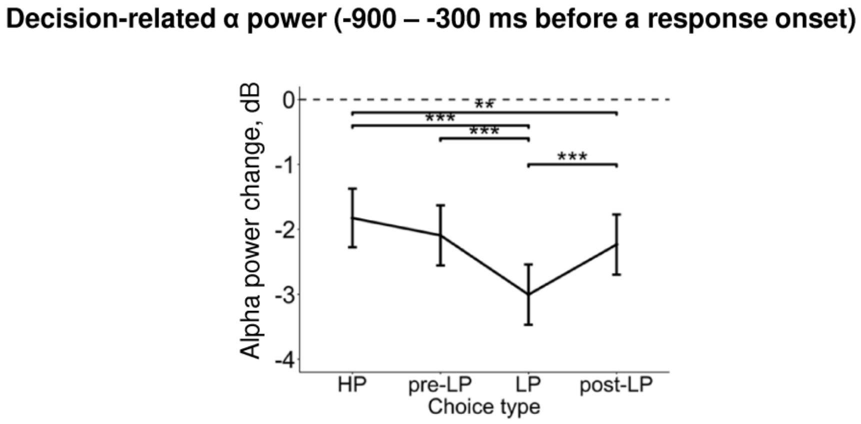

Supplementary Figure S3. Enhanced decision-related α-power suppression (α-ERD) distinguishes explorative LP in a pattern similar to that for β-power suppression (β-ERD). Decision-related α-band power change was averaged within -900 - -300 ms relative to response, over the cluster of sensors marked by colored rings in Figure 3A. The α power change for each choice type shows averages across sensors per the cluster marked. Points and error bars on graphs represent M ± SEM in single trials in all subjects.

** – p<0.01, *** – p<0.001 (LMM, Tukey HSD test).

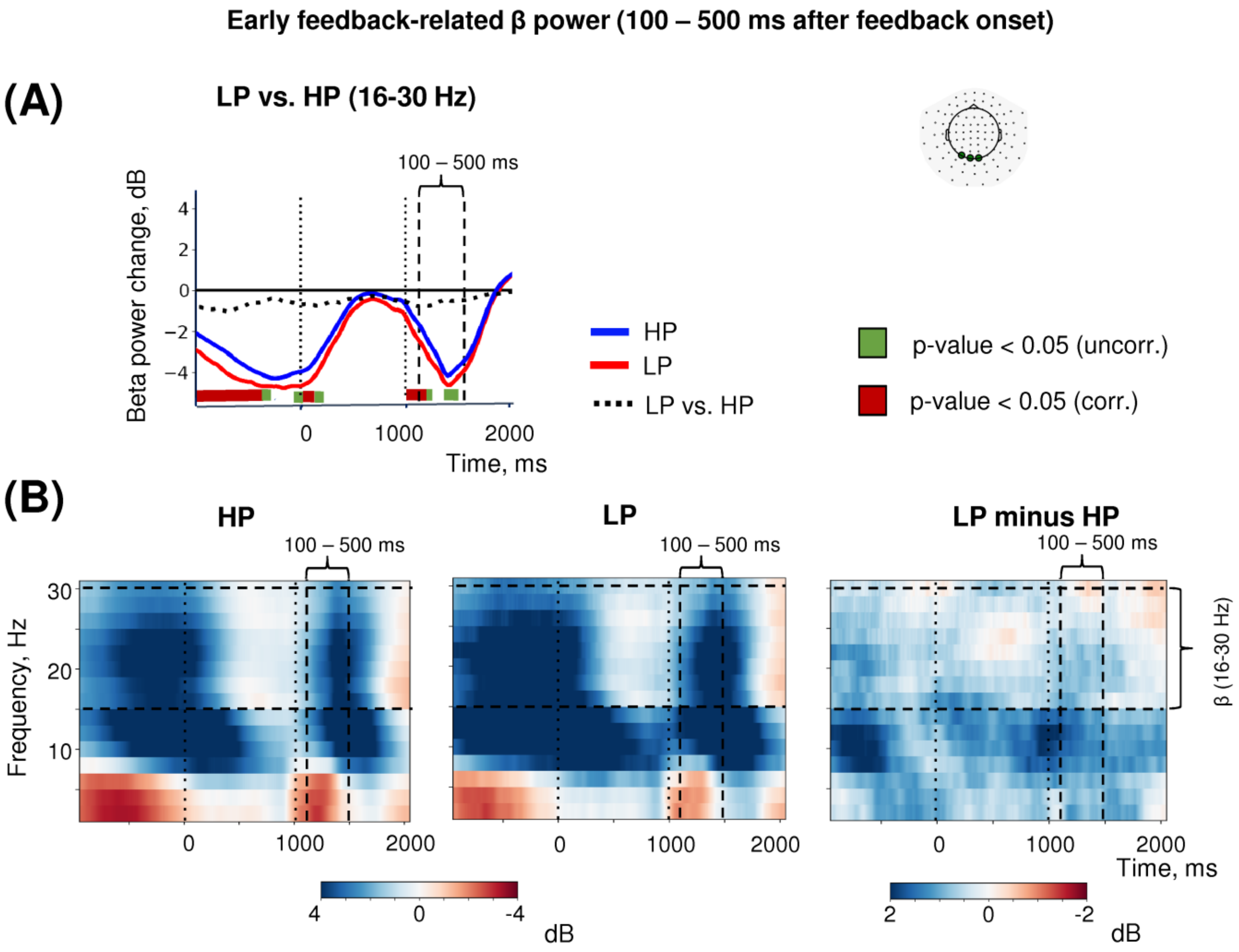

Supplementary Figure S4. Additional time-frequency analysis for the early feedback effect.

(A) Timecourses of β-band power averaged over three most significant sensors (shown in the inset at the top). Each graph represents two timecourses and the difference between them. Lines at the bottom of each graph indicate significance of the difference (t-test, $p < 0.05$; green: uncorrected; brown: FDR corrected over time points).

(B) Event-related spectral perturbations of MEG power averaged within the same three sensors obtained in the main LMM analysis.

'0' on the timeline corresponds to response, and '1000' – to feedback onset. '100 – 500 ms' denotes respective time window relative to feedback onset.

Supplementary Table S1. Choice Types and Overall Behavioral Statistics

Choice type	Trials[¥]: Previous → **Current** → Next	Number of trials	% of trials	RT, ms (*M* ± *SD*)
HP	A → **A** → A	3148	64.4	1383 ± 88
pre-LP	A → **A** → DA	600	12.3	1502 ± 96
LP	A → **DA** → A	539	11.0	1806 ± 95
post-LP	DA →**A** → A	602	12.3	1514 ± 97

[¥] A – advantageous choice, DA – disadvantageous choice.

Supplementary Table S2. LMM statistics for the response time

	**df1**	**df2**	**F**	**p**
**Choice type**	**3**	**3519.35**	**17.09**	**<0.001*****
Previous feedback	1	3519.89	1.85	0.174
**Choice type x Previous feedback**	**3**	**3515.64**	**4.72**	**0.003****

** – p<0.01, *** – p<0.001.

Supplementary Table S3. LMM statistics for the effects of choice types, feedback valence, and their interaction for beta power change averaged across the pre-specified sensors and time windows based on the previous LMM analysis results[¥]

Effect	Factor	df1	df2	F	p
Decision-related effect	**Choice type[†]**	**3**	**4324.4**	**28**	**<0.001*** **
Early feedback-related effect	**Choice type[†]**	**3**	**4317.9**	**13.9**	**<0.001*** **
	Feedback	1	4316.5	0.02	0.874
	Choice type x Feedback	3	4316.9	1.65	0.176
Late feedback-related effect (posterior cluster)	**Choice type**	**3**	**4318.6**	**2.8**	**0.037***
	**Feedback**	**1**	**4316.8**	**13.7**	**<0.001*** **
	**Choice type x Feedback[†]**	**3**	**4317.4**	**13.3**	**<0.001*** **
Late feedback-related effect (anterior cluster)	**Choice type**	**3**	**4322.8**	**9.78**	**<0.001*** **
	**Feedback**	**1**	**4316.9**	**37.3**	**<0.001*** **
	**Choice type x Feedback[†]**	**3**	**4319.1**	**11**	**<0.001*** **

[¥] LMM statistics were computed to ensure that the effects obtained for beta power at the level of single sensors and time bins survived after averaging beta values across space and time and should not be considered in isolation from the previous results.

[†] Significance is a consequence of the procedure used to define time intervals and clusters of sensors (cf. Figure 2 in the main text).

* – p<0.05, *** – p<0.001 (Tukey HSD).
